# Predictors of admission to an assertive outreach service for psychosis in Lebanon

**DOI:** 10.1192/j.eurpsy.2024.456

**Published:** 2024-08-27

**Authors:** G. Kassir, S. El Hayek, R. Charara, M. Cherro, H. Itani, J. El Khoury

**Affiliations:** ^1^Psychiatry, American University of Beirut, Beirut, Lebanon; ^2^Psychiatry and Behavioral Sciences, University of Miami Miller School of Medicine, Miami; ^3^Center of Behavvioral Health, Cleveland Clinic, Cleveland, United States; ^4^Psychiatry and Behavioral Health, American Hospital Dubai, Dubai, United Arab Emirates

## Abstract

**Introduction:**

Schizophrenia is a chronic, debilitating mental illness that contributes significantly to the global burden of disease. Assertive outreach treatment for patients with schizophrenia and psychotic disorders has been implemented to improve treatment adherence and outcomes.The suitability of this model of care outside the western context has not been fully established. The Psychosis Recovery Outreach Program (PROP),staffed by a multidisciplinary team that applies principles of early intervention and assertive outreach, was initiated in February 2016 at a leading psychiatric facility in Lebanon.

**Objectives:**

The aim of this study is to identify and analyze clinical and demographic variables associated with patient enrollment in PROP, out of a typical clinical population attending a psychiatric outpatient department.

**Methods:**

This retrospective study included patients above 18 y.o. at time of first point of care with a primary diagnosis of psychosis according to the International Classi-fication of Diseases 10 (ICD-10), and who presented to the outpatient psychiatry department at the American University of Beirut Medical Center (AUBMC) and were following up in PROP. We collected twelve-month data and used logistic regression models to identify predictor variables for enrollment in the service compared to those receiving standard treatment.

**Results:**

In total, 45 patients participated in the study. Patients were mostly males (77.8%), younger than 39 years (80%), of college or higher education (68.2%), and diagnosed with schizophrenia (46.7%) or schizoaffective disorder (48.9%). About one-quarter (22.7%) had a comorbid cannabis use disorder. A majority received more than one oral antipsychotic (75.6%) while half (51.1%) were maintained on a long-acting injectable (LAI) antipsychotic.The following variables were significant predictors of enrollment in PROP: having a comorbid cannabis use disorder (OR 2.83 [1.25 – 6.37]), being prescribed a LAI antipsychotic (OR 9.99 [4.93-20.24]) or more than one oral antipsychotic (OR 4.57 [2.22-9.39]), visiting the emergency department more than once (OR 8.7 [2.64-28.68]), and admission to the psychiatry unit (OR 13.91[3.17-60.94]). In addition, those following up in PROP were younger and less likely to be in the oldest age group (over 54 years) [OR 0.11 (0.01-0.93)], less likely to be females (OR0.39 [0.18-0.81]), and less likely to be diagnosed with “other psychotic disorder” as com-pared to schizophrenia (OR 0.14 [0.03 – 0.62]).

**Conclusions:**

PROP was the first community treatment program to use the principles of assertive outreachin Lebanon. Our findings highlight that the assertive out-reach model of care is applicable to its target population in the context of psychiatric care in Lebanon, namely young individuals with psychosis, higher comorbidities and a severe course of illness.

**Disclosure of Interest:**

None Declared

